# Refrigerated storage of L-PRF membranes: anti-bacterial activity, cytokine release, and *in vitro* keratinocyte effects—an *in vitro* pilot study

**DOI:** 10.3389/froh.2026.1905496

**Published:** 2026-08-04

**Authors:** Jize Yu, Ana Castro Sarda, Naiera Zayed, Wim Teughels, Marc Quirynen

**Affiliations:** Department of Oral Health Sciences, Periodontology and Oral Microbiology (P&OM), KU Leuven, Leuven, Belgium

**Keywords:** autogenous grafts, cell- and tissue-based therapy, leukocyte- and platelet-rich fibrin, platelet concentrates, tissue engineering

## Abstract

**Aims:**

This *in vitro* study aimed to evaluate the effect of storage, for up to 4 weeks, on the degradation kinetics and bioactivity of leukocyte- and platelet-rich fibrin (L-PRF) membranes.

**Methods:**

L-PRF membranes obtained from 8 volunteers were stored in L-PRF exudate at 4°C for 1, 2, or 4 weeks. Their antibacterial properties against the main periodontal pathogens, release levels of key growth factors and inflammatory cytokines, and regenerative effects on human oral keratinocytes (HOKs) were compared to fresh L-PRF.

**Results:**

The inhibitory effects of L-PRF exudate varied among the tested pathogens and gradually decreased during storage. Growth factors released from L-PRF membranes exhibited different degradation kinetics, with VEGF demonstrating an early reduction within the first week, whereas PDGF-AB and TGF-*β*1 remained relatively stable until 2 weeks. Interleukin (IL)-8, IL-1b, and IL-6 were detectably released from L-PRF membranes, predominantly in fresh samples, and decreased within the first week. Conditioned medium derived from stored L-PRF membranes significantly promoted HOK metabolic activity and wound healing *in vitro*, without significant reduction over the 4-week storage period.

**Conclusion:**

Refrigerated storage preserved some L-PRF functions for up to 4 weeks in this *in vitro* study, but preservation was incomplete and function-specific, with the clearest losses in antibacterial activity, VEGF release, and inflammatory cytokine release.

## 1 Introduction

Leukocyte- and platelet-rich fibrin (L-PRF) is a second-generation platelet concentrate, succeeding platelet-rich plasma (PRP), that has been widely applied in oral and maxillofacial regenerative therapies (1–6). Its dense fibrin scaffold serves as both a physical barrier and a reservoir of platelets, leukocytes, and bioactive molecules that promote wound healing (3, 7). These combined functions enable L-PRF to create a microenvironment that can accelerate tissue repair while suppressing microbial growth (8, 9), which is particularly relevant for oral and some extraoral wounds where microbial colonization may delay or compromise healing (7). However, the requirement for an L-PRF-specific centrifuge for preparation and the need for immediate application after preparation constrain its clinical flexibility, particularly in conditions requiring repeated interventions (e.g., diabetic foot ulcers, flap surgery in several quadrants at different appointments). Preparing a large batch of L-PRF membranes at the first appointment and storing them for the following sessions would reduce the need for repeated blood collection and simplify the workflow of the treatment. This approach may be particularly beneficial for home healthcare providers who lack access to an L-PRF specific centrifuge or the expertise to perform venipuncture. Given the distinct composition and clinical applications of L-PRF (extraction socket filling/sinus fill/wound dressing), current storage protocols for human blood products are hardly suitable for preserving the regenerative capacity of L-PRF membranes.

Recent investigations by our research group demonstrated that L-PRF membranes maintained morphological integrity and tensile properties for up to 4 weeks storage at 4°C (10). However, a progressive decline in leukocyte viability was observed during storage, which has been associated with the accumulation of reactive oxygen species (ROS) and metabolic waste in other blood products, ultimately compromising the functions of leukocytes (e.g., chemotaxis (11) and phagocytosis (12)). The observed impairment of leukocytes during storage then raised concerns about the functional integrity of the stored L-PRF membranes, particularly regarding their antibacterial activity against periodontal pathogens and the release profile of pro-inflammatory cytokines that may interfere with the regeneration process. On the other hand, platelets entrapped within the fibrin scaffold represent the primary source of growth factors in L-PRF. During storage, platelet degranulation (13, 14) and the gradual degradation of released growth factors may alter their release kinetics (15–19), potentially affecting the regenerative properties of stored L-PRF membranes. Therefore, the influence of storage on growth factor release can be more complex compared to other blood products and requires further investigation.

Taken together, this study aimed to evaluate the antibacterial activity, release of growth factors and inflammatory cytokines, as well as *in vitro* regenerative effects on human oral keratinocytes (HOKs) of stored L-PRF membranes (for up to four weeks), using freshly prepared membranes as the reference. We hypothesized that refrigerated storage would preserve L-PRF biological functionality for a limited period, followed by a gradual decline during prolonged storage. This work complements our recent integrity observations of L-PRF membranes after storage (10), by providing functional evidence supporting the clinical applicability of stored L-PRF membranes.

## 2 Methods

### 2.1 Volunteer selection

This was an *in vitro* pilot study using L-PRF membranes obtained from eight systemically and periodontally healthy adult volunteers, after the approval of the ethical committee of KU Leuven (No. S58789, third amendment). The exclusion criteria included: anticoagulant medication or antibiotics 3 months prior to the study, pregnancy or lactation, and history of any active systemic infection or psychological constraints. Participants with conditions or medications known to interfere with platelet function or wound healing were also excluded. All procedures were performed according to the Helsinki Declaration and the regulations of the University Hospital. An informed consent was obtained from all subjects after having explained the purpose of the study.

### 2.2 L-PRF preparation & storage

Forty milliliters of venous blood from each volunteer were collected in four silica-coated plastic vacuum tubes without anticoagulant (10 mL blood in each tube) and immediately centrifuged at 408 g for 12 min (IntraSpin, Boca Raton, FL, USA). The L-PRF clots were retrieved from the tubes and after gentle removal of the red blood cells, they were compressed into membranes using the Xpression Box® (Intra-Lock, Boca Raton, FL, USA). All procedures preparing L-PRF membranes were performed under aseptic conditions using sterile instruments to minimize contamination. All L-PRF membranes obtained from a single volunteer were stored together in L-PRF exudate (liquid from L-PRF clots after compression) in a dedicated sterile tube at 4°C until analysis. At each predefined time point (7, 14, and 28 days), one membrane was retrieved for conditioned medium preparation.

### 2.3 L-PRF conditioned medium preparation

**Figure 1:**
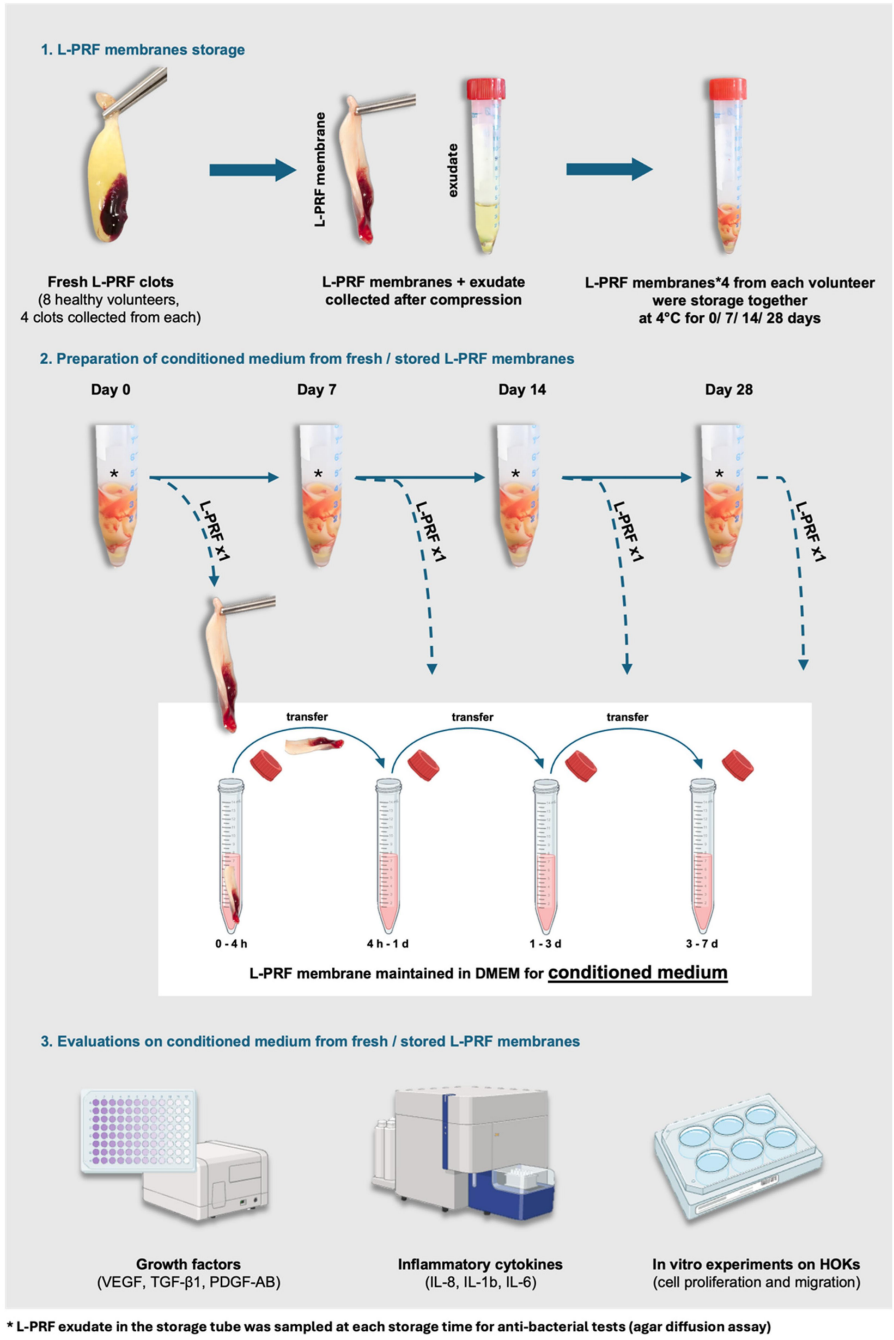
Schematic illustration of L-PRF membrane preparation, refrigerated storage, and in vitro evaluation of storage-related changes in antibacterial activity, growth factor and cytokine release, and human oral keratinocyte proliferation and migration.

The L-PRF conditioned medium was prepared following the protocol described by Castro and co-workers (20), fresh L-PRF membrane (less than 20 min after preparation) as well as 7-, 14-, and 28-day stored membranes were placed in 15 mL centrifuge tubes supplemented with 5 mL of Dulbecco's Modified Eagle medium (DMEM, Sigma-Aldrich, St. Louis, Missouri, United States) for incubation without supplemented antibiotics or fetal bovine serum. Each L-PRF membrane was transferred to a new tube with 5 mL of fresh DMEM after 4 h and subsequently after 1, 3, and 7 days. The conditioned medium from fresh or stored L-PRF membranes at different intervals (0–4 h, 4 h–1 d, 1 d–3 d, 3 d–7 d) was immediately centrifuged to remove any residue and then cryopreserved at −80°C for further assays (Figure 1). Conditioned media from each donor were analyzed separately and were not pooled. Accordingly, *n* = 8 represents independent donors rather than the individual membrane.

### 2.4 Antibacterial activity (agar diffusion assay)

The antibacterial activity of exudate from L-PRF membranes after storage was evaluated against key periodontal pathogens via an agar diffusion assay. *Prevotella intermedia ATCC 25611, Porphyromonas gingivalis ATCC 33277, Fusobacterium nucleatum DSM 20482, and Aggregatibacter actinomycetemcomitans ATCC 43718* were maintained on blood agar plates (supplemented with 5.0 mg/L hemin, 1.0 mg/L menadione, and 5% sterile horse blood) under anaerobic conditions except for *A. actinomycetemcomitans* which was maintained at 5% CO_2_*.* Liquid cultures and bacterial suspensions were prepared using Brain-Heart Infusion Broth (BHI). 10 µL of L-PRF exudate were collected from the storage tube at each time point (7/ 14/ 28 days), followed by freezing for future tests. The exudate from fresh/stored L-PRF was then loaded on standardized sterile round paper disks (diameter = 6 mm) then placed on blood agar plates seeded with 80 ml of either *A. actinomycetemcomitans*, *F. nucleatum*, *P. gingivalis*, or *P. intermedia* suspensions. (Bacteria were prepared by growing periodontal pathogens to an OD_600_ corresponding to ∼1 × 10^8^ CFU/mL, then diluted into a working concentration of 1 × 10^7^ CFU/mL based on established OD_600_-CFU calibration curves (16, 17).) Each donor contributed one agar plate with four paper disks (representing four storage time points) for each bacterium. After 5-days of incubation under the appropriate incubation conditions for each bacterium, standardized pictures were taken (DSC-HX400V, SONY, Japan) with a calibration ruler beside. The bacterial growth inhibition zone around each paper disk was calculated using ImageJ (v2.14.0/1.54f) (The measured area of the PBS control corresponds to the baseline disk area and therefore represents the absence of bacterial inhibition).

### 2.5 Growth factor release (enzyme-linked immunosorbent assay)

The concentrations of vascular endothelial growth factor (VEGF), transforming growth factor beta-1 (TGF-β1), and platelet-derived growth factor-AB (PDGF-AB) in the conditioned medium from fresh/stored L-PRF membranes were measured using commercially available enzyme-linked immunosorbent assay (ELISA) kits (R&D Systems Europe, Abingdon, UK) following the manufacturer's instructions. Each sample was analyzed in duplicate with a microplate reader (Multiskan Ascent, Rev 1.2, Thermo Electron Corporation, Vantaa, Finland).

### 2.6 Inflammatory cytokine release (cytometric bead array assay)

Cytokine release was quantified using a BD™ Cytometric Bead Array (CBA) Human Inflammatory Cytokines Kit (BD Biosciences, San Jose, CA, USA), according to the manufacturer's instructions. Briefly, 50 μL of each L-PRF membranes conditioned medium was incubated with the capture bead mixture and the phycoerythrin (PE)-conjugated detection reagent for 3 h at room temperature in the dark. Following incubation, samples were washed and resuspended in 300 μL of wash buffer. Data acquisition was performed on a BD FACSVerse cytometer (BD Biosciences, Belgium), and cytokine concentrations (IL-1b, IL-6, IL-8, IL-10, TNF, IL-12p70) were calculated using the Software FlowJo 10.9.0.

### 2.7 Cell metabolic activity (XTT assay)

The effect of L-PRF membranes conditioned media on cell metabolic activity, as an indirect indicator of cell proliferation, was evaluated using the XTT Cell Proliferation Kit (Sigma-Aldrich, St. Louis, Missouri, United States). Human oral keratinocytes were seeded in 96-well plates at a density of 3 × 10^3^ cells/well and allowed to adhere overnight. Subsequently, the culture medium was replaced with the conditioned medium derived from fresh/stored L-PRF membranes or with keratinocyte medium (KM)/DMEM as the control group. After 24 h of co-culture, the conditioned medium was replaced with 100 μL serum-free DMEM. Thereafter, 50 μL XTT working solution was added to each well, and following an 8 h incubation, absorbance was measured at 450 nm using a microplate reader, with 650 nm as the reference wavelength.

### 2.8 *In vitro* wound closure (scratch assay)

An *in vitro* wound closure model was performed to detect the HOK proliferation and migration under the L-PRF membranes conditioned medium. In 6-well plates, 2 × 10^5^ HOK cells/well were seeded with serum-free DMEM for 24 h before the wound healing assay. A 1 mL pipette tip was used to create a cross scratch, followed by washing the cells with phosphate-buffered saline (PBS) and replacing the medium with the L-PRF conditioned medium. A microscope was used to record the scratch area immediately and after 24 h of healing. The proportion of wound closure was calculated using Software ImageJ.

### 2.9 Statistical analysis

Data are presented as mean ± standard deviation (SD). Statistical analyses were performed using GraphPad Prism 10.0 (GraphPad Software, San Diego, CA, USA). To account for the repeated-measures design, a linear mixed-effects model was fitted with storage time as a fixed effect and subject as a random effect. The Shapiro–Wilk test was used to assess the normality of residuals. Pairwise comparisons between storage conditions were adjusted for multiple testing using Tukey's method. Statistical significance was set at *p* < 0.05.

## 3 Results

### 3.1 Baselines of volunteers

Eight volunteers (4 female and 4 male) participated in this study (Table 1). The mean age was 60.4 ± 11.7 years (range 42–77 years). No complications during blood collection were reported.

**Table 1:** Baselines of volunteers.

Volunteer	Baselines				
	Gender	Age (year)	Comorbidities	Smoking history	Medication
1	Male	61	No	No	No
2	Female	52	Hypertension	No	ACE inhibitor
3	Female	67	No	No	No
4	Male	63	Hypertension	No	No
5	Female	50	No	No	No
6	Female	42	No	No	No
7	Male	77	Hypertension	Ex	ACE inhibitor, statin
8	Male	71	No	Ex	No

ex, ex-smoker; ACE, angiotensin-converting enzyme.

### 3.2 Antibacterial activity (agar diffusion assay)

**Figure 2:**
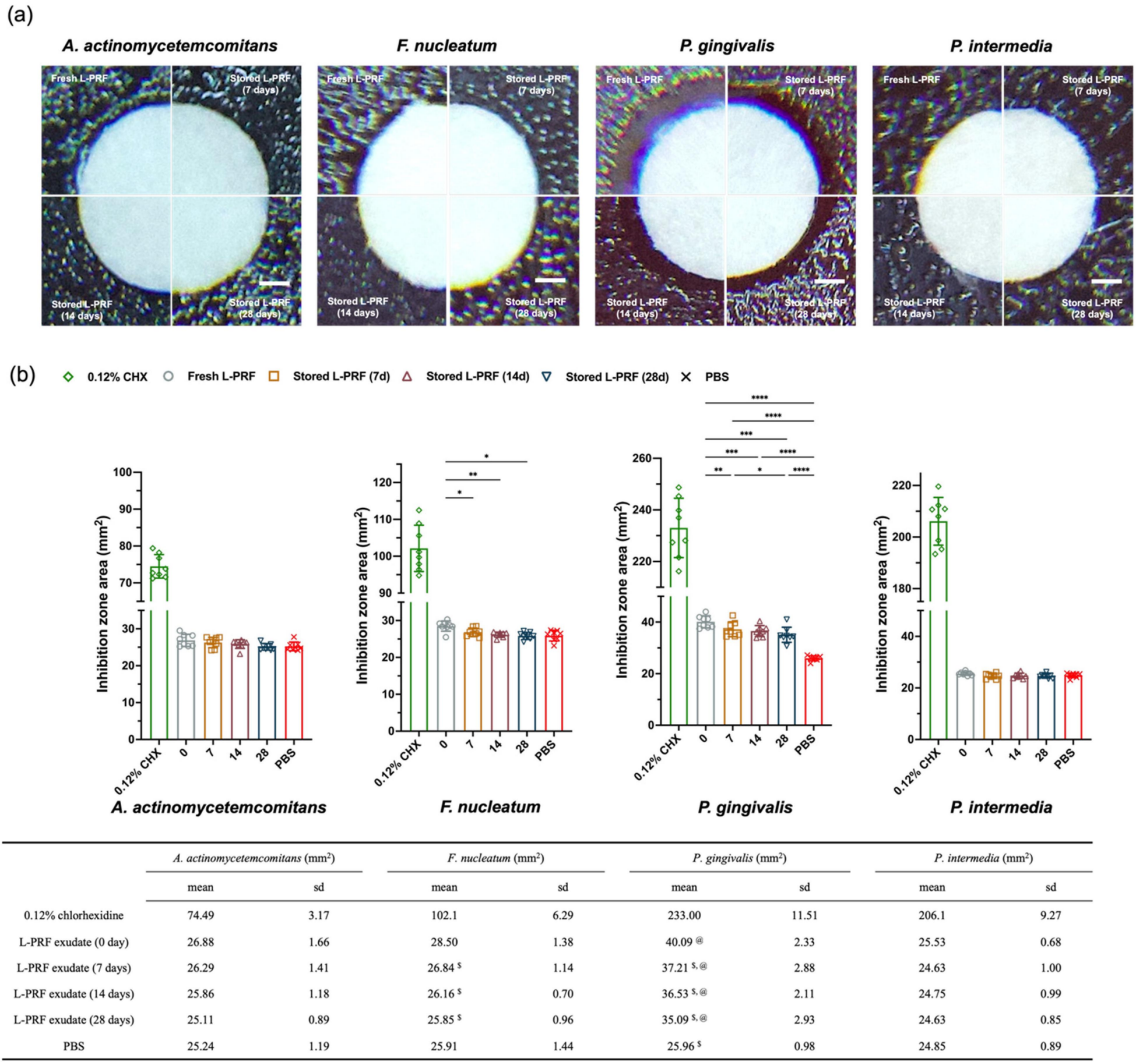
Anti-bacterial capacity of L-PRF exudate against common periodontal pathogens during their storage. **(a)** Representative inhibition zone on blood agar plates with common periodontal pathogens. Scale bar: 2 mm. **(b)** Quantitative analysis of the area of inhibition zone. (*n* = 8). Statistical analysis was performed using a linear mixed-effects model with Tukey's test. *p < 0.05, **p < 0.01, ***p < 0.001, ****p < 0.0001, $ = statistically significant difference compared to fresh L-PRF exudate; @ = statistically significant difference compared to PBS (negative control). PBS, phosphate-buffered saline; sd, standard deviation; CHX, chlorhexidine. The measured area of the PBS control corresponds to the disk area itself and therefore represents the absence of bacterial growth inhibition*.*

The antibacterial activity of exudate from fresh L-PRF membranes exhibited considerable variation among different periodontal pathogens, with significant inhibition observed of *P. gingivalis*, while negligible inhibition was observed against *A. actinomycetemcomitans*, *F. nucleatum, and P. intermedia* (Figure 2a). During the 4-week storage, the inhibitory zone of L-PRF exudate against *P. gingivalis* significantly decreased from (40.1 ± 2.3 mm^2^) on day 0 to (37.2 ± 2.9 mm^2^, *p* < 0.01) on day 7, reaching (36.5 ± 2.1 mm^2^, *p* < 0.001) and (35.1 ± 2.9 mm^2^, *p* < 0.001) on day 14 and day 28, respectively. However, inhibition against *P. gingivalis* was maintained throughout the 4-week storage and statistical significance was achieved in all comparisons between storage groups and PBS control (corresponding to the absence of bacterial inhibition, *p* < 0.0001). On the other hand, fresh L-PRF exudate exhibited higher inhibitory effects against *A. actinomycetemcomitans and F. nucleatum* than PBS control, even though no statistical significance was achieved (Figure 2b).

### 3.3 Release of growth factors (enzyme-linked immunosorbent assay)

**Figure 3:**
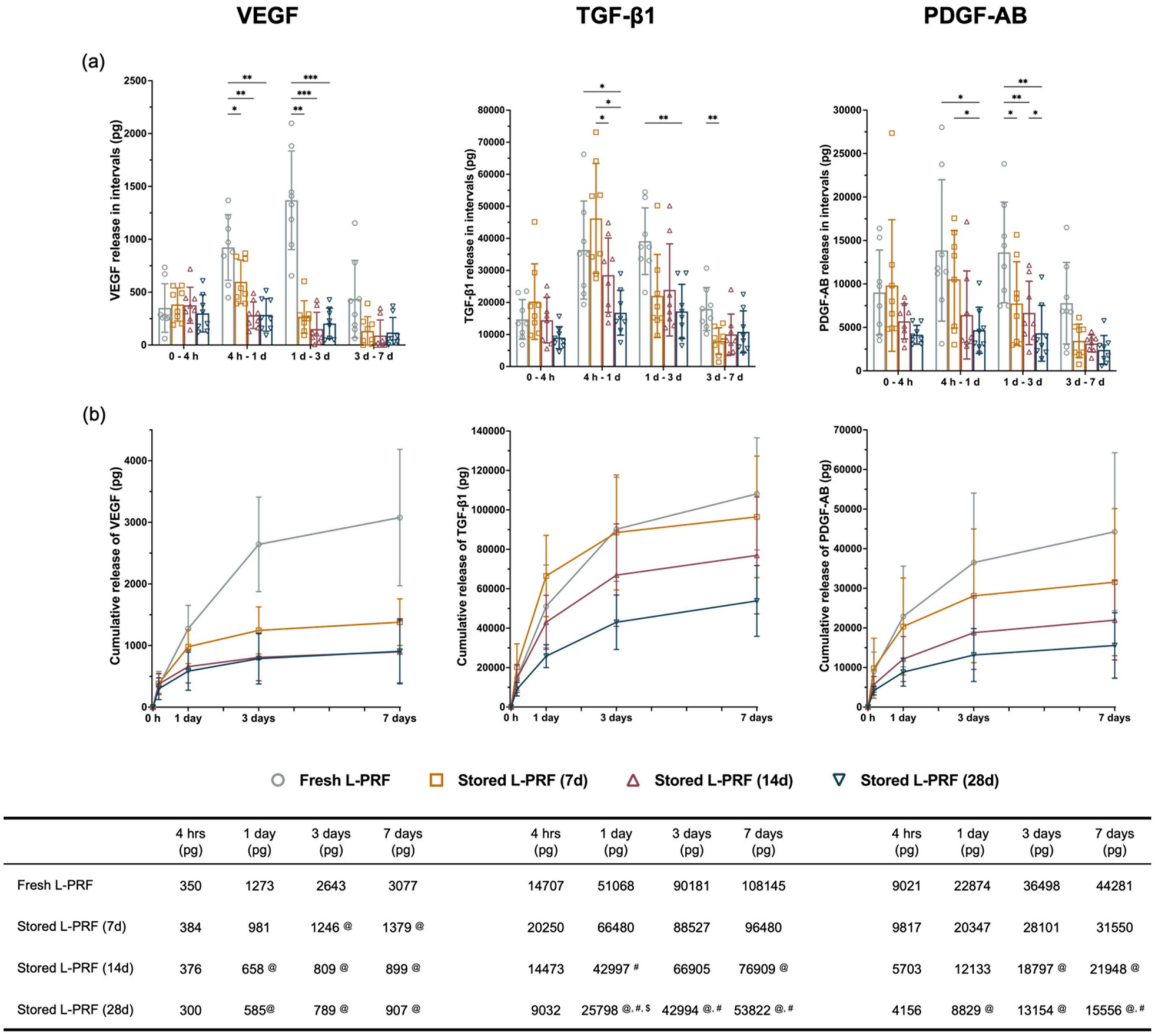
Growth factor releases from fresh/stored L-PRF membranes. **(a)** VEGF, TGF-*β*1, and PDGF-AB release in intervals over 7 days (*n* = 8). **(b)** Cumulative release of VEGF, TGF-*β*1, and PDGF-AB over 7 days (*n* = 8). Statistical analysis was performed using a linear mixed-effects model with Tukey's test. *p < 0.05, **p < 0.01, ****p < 0.0001, @ = statistically significant difference compared to fresh L-PRF; # = statistically significant difference compared to stored L-PRF (7d); $ = statistically significant difference compared to stored L-PRF (14d). VEGF, vascular endothelial growth factor; TGF-*β*1, transforming growth factor beta-1; PDGF-AB, platelet-derived growth factor-AB.

All the tested growth factors were continuously released from stored L-PRF for up to 7 days, with a faster release overall being within the first 24 h, followed by a gradual deceleration in release kinetics. Compared to fresh L-PRF, stored L-PRF exhibited an earlier peak in release kinetic, resulting in a burst release in some growth factors (e.g., 7-day stored L-PRF demonstrated higher release levels in all tested growth factors within the initial 4 h and even higher release level in TGF-*β*1 within the initial 24 h). The early burst of release led to reduction in growth factor release to some extent at later release intervals, which was especially pronounced with VEGF, significantly reducing after the first 4 h release in 7-day stored L-PRF. However, the statistical significances in TGF-*β*1 and PDGF-AB release mainly reached at the 4 h–1 d and 1–3 d interval between fresh L-PRF and 28-day stored L-PRF (Figure 3a).

The cumulative release of growth factors was also calculated (Figure 3b). The highest amount of VEGF over 7 days was released by fresh L-PRF membranes (3,077 pg), with a significantly higher level than all stored membranes (3,077 vs. 1,379, 899, and 907 pg, for 7-day (*p* < 0.05), 14-day (*p* < 0.01), and 28-day (*p* < 0.01) stored L-PRF membranes respectively. For TGF-*β*1, the highest amount over 7 days was also achieved by fresh L-PRF membranes, significantly higher than 14- and 28-day stored L-PRF (108,145 vs. 76,909 pg, *p* < 0.05 and 53,822 pg, *p* < 0.01). Similarly, the highest level of PDGF-AB was again released by fresh membranes, with significant differences compared to the 14-day and 28-day stored membranes (44,281 vs. 21,948 pg, *p* < 0.05 and 15,556 pg, *p* < 0.05). Overall, refrigerated storage predominantly reduced cumulative VEGF release, with only 29.5% of VEGF remained after 28 days of storage, whereas 49.8% of TGF-*β*1 and 35.1% of PDGF-AB were preserved.

### 3.4 Cytokine release (cytometric bead array assay)

**Figure 4:**
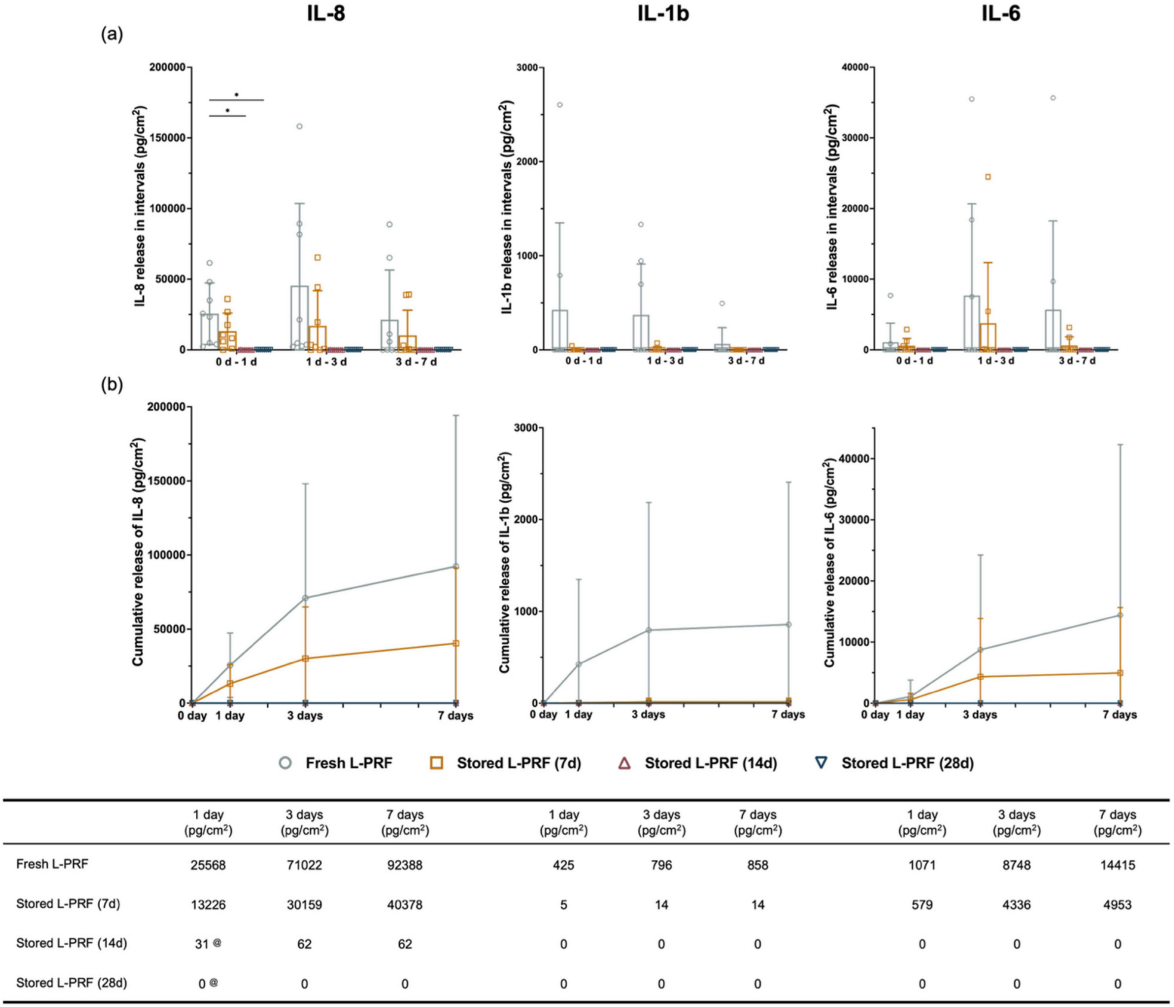
Inflammatory releases from fresh/stored L-PRF membranes. **(a)** IL-8, IL-1b, and IL-6 release in intervals over 7 days (*n* = 8). **(b)** Cumulative release of IL-8, IL-1b, and IL-6 over 7 days (*n* = 8). Statistical analysis was performed using a linear mixed-effects model with Tukey's test. *p < 0.05, @ = statistically significant difference compared to fresh L-PRF. IL, Interleukin. * According to the manufacturer's instructions for the cytokine assay kits, values below the limit of detection were recorded as zero (IL-8 <8.6 pg/cm^2^, IL-1b <4.2 pg/cm^2^, and IL-6 <6.0 pg/cm^2^).

Of the cytokines assessed (IL-8, IL-1b, IL-6, IL-10, TNF, IL-12p70), only IL-8, IL-1b, and IL-6 exhibited detectable releases. Although an overall decline in cytokine release was observed with increasing storage duration, these patterns should be interpreted cautiously because many samples yielded concentrations below the assay detection limit, resulting in substantial inter-donor variability (Figure 4). Statistically significant differences were only found in IL-8 between fresh L-PRF and L-PRF stored for 14 days (*p* < 0.05) and 28 days (*p* < 0.05). Throughout the storage, most of the tested inflammatory cytokines were released within the first 3 days, with the fresh membranes exhibiting the highest cumulative release (92,388 pg/cm^2^ for IL-8, 858 pg/cm^2^ for IL-1b, and 14,415 pg/cm^2^ for IL-6), while after 7 days of storage, IL-8, IL-1b, and IL-6 showed rapid reduction in cumulative release (40,378 pg/cm^2^ for IL-8, 14 pg/cm^2^ for IL-1b, and 4,953 pg/cm^2^ for IL-6). After 2 and 4 weeks of storage, inflammatory cytokines could be rarely detected.

### 3.5 Cell metabolic activity (XTT assay)

**Figure 5:**
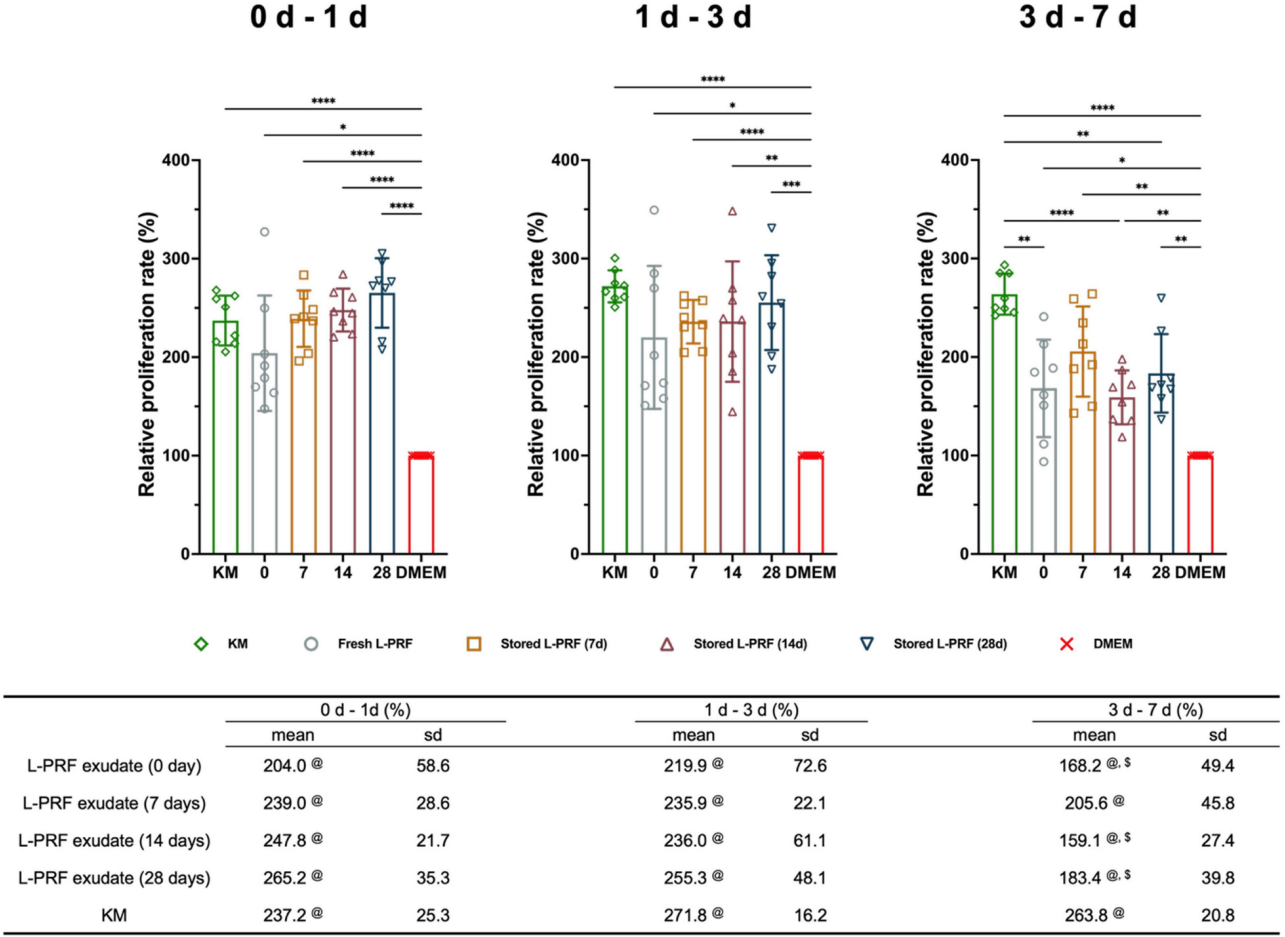
XTT assays on human oral keratinocytes cultured with conditioned medium from fresh/stored L-PRF (*n* = 8). Statistical analysis was performed using a linear mixed-effects model with Tukey's test. *p < 0.05, **p < 0.01, ***p < 0.001, ****p < 0.0001, @ = statistically significant difference compared to DMEM (negative control); $ = statistically significant difference compared to KM (positive control). KM, keratinocyte medium; DMEM, Dulbecco's Modified Eagle medium; sd, standard deviation.

Fresh L-PRF membranes significantly promoted HOKs' metabolic activity by 204%, 220%, and 168% compared to DMEM (basic medium used for preparing L-PRF conditioned medium, negative control) at 0 d–1 d, 1 d–3 d, and 3 d–7 d time intervals, respectively (*p* < 0.05). In comparison to fresh L-PRF, stored L-PRF after varying durations exhibited minimal reduction in the enhancement of HOKs' metabolic activity at all time intervals (Figure 5), demonstrating statistically significant differences to DMEM at all stages and similar levels with keratinocyte medium at early release stages. However, at the late release stage (3 days to 7 days), the promotion of L-PRF (both fresh L-PRF and stored L-PRF) began to decline and exhibited significant differences compared to KM (*p* < 0.01).

### 3.6 *In vitro* wound closure (scratch assay)

**Figure 6:**
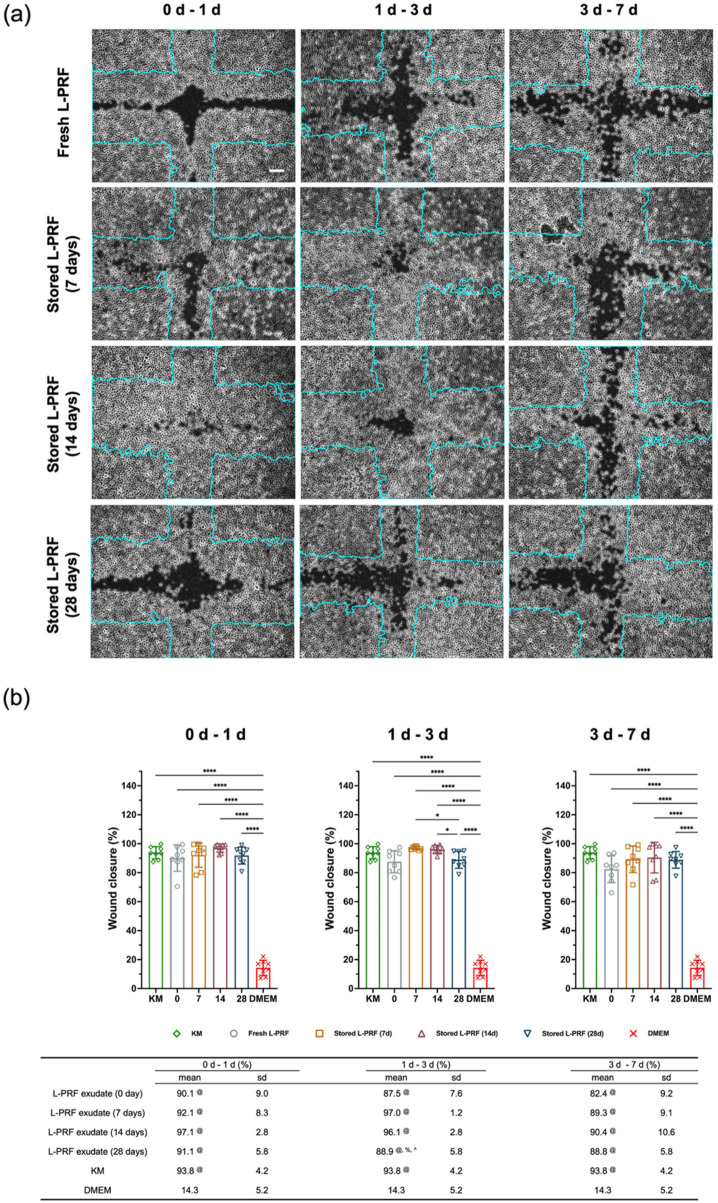
Wound healing assays on human oral keratinocytes cultured with conditioned medium from fresh/stored L-PRF (*n* = 8). **(a)** Representative microscopic view of the “wound closure” before (outlines in cyan) and after being cultured with L-PRF conditioned medium. Scale bar: 100 μm. **(b)** Proportion of wound closure after a 24 h culture with L-PRF conditioned medium. (*n* = 8). Statistical analysis was performed using a linear mixed-effects model with Tukey's test. *p < 0.05, **p < 0.01, ****p < 0.0001. @ = statistically significant difference compared to DMEM (negative control); % = statistically significant difference compared to stored L-PRF (7d); ^ = statistically significant difference compared to stored L-PRF (14d). KM, keratinocyte medium; DMEM, Dulbecco's Modified Eagle medium; sd, standard deviation.

*In vitro* wound healing assay indicated that the proliferation and migration of HOKs was significantly enhanced by fresh/stored L-PRF membranes (Figure 6a). The closure of the original wound area significantly increased from 14.3% in DMEM to 90.1%, 87.5%, and 82.4% in the fresh L-PRF group at 0 d–1 d, 1 d–3 d, and 3 d–7 d time intervals, respectively (Figure 6b, *p* < 0.0001). Interestingly, HOKs cultured with stored L-PRF exhibited slightly higher closure proportions compared to the fresh L-PRF, even though no statistical significance was achieved. During the 28-day storage period, stored L-PRF maintained comparable levels of activity *in vitro* in promoting HOKs wound closure with KM, demonstrating significant superiority over DMEM.

## 4 Discussion

This pilot study evaluated the time-dependent changes in the biological functions of L-PRF membranes after refrigerated storage. The findings demonstrate that storage resulted in a progressive and function-specific decline in L-PRF bioactivity rather than uniform preservation of all biological properties. The bioactivity of the stored membranes was evaluated in terms of antibacterial properties, release of growth factors and inflammatory cytokines, and their *in vitro* regenerative effects on human oral keratinocytes (HOKs). In this study, the storage condition was set to reflect the intended applications in home healthcare. The temperature was selected at 4°C as World Health Organization guidelines for human whole blood storage (21) and household refrigerators' setting. The storage medium was chosen based on the consideration of avoiding the introduction of exogenous preservation agents (e.g., DMSO) and full use of the exudate after the preparation of L-PRF membrane. Therefore, the storage condition in this study can be achieved in clinical scenario.

L-PRF membrane exudates demonstrated their most pronounced antibacterial activity against *P. gingivalis* and only modest inhibitory effects against the other tested pathogens, aligning with the findings reported by Castro et al. (9) and Rodríguez Sánchez et al. (22) This antimicrobial activity of L-PRF exudate against *P. gingivalis* was partly attributed to the reactive oxygen species (ROS) released from L-PRF membranes (22). L-PRF exudate retained a significant inhibitory effect against *P. gingivalis* after 28 days of storage, whereas *A. actinomycetemcomitans*, *P. intermedia* and *F*. *nucleatum* exhibited higher resistance against stored L-PRF exudate, which might be explained by their species-specific sensitivity to ROS (23–25) and higher aggregation tendencies to physically protect themselves from the exposure to antimicrobial agents (26). Notably, the susceptibility is not only strain- but also context-dependent, and controversial results might be obtained under other specific assay conditions, e.g., L-PRF membrane preparation protocol (27) and biofilm communities (22). Because only exudates were tested, the present assay primarily reflects the antibacterial activity of soluble components released from L-PRF membranes rather than direct leukocyte-mediated antibacterial effects within the membrane itself. Moreover, these findings should not be interpreted as indicating that L-PRF is intended to replace conventional antiseptics such as chlorhexidine. Rather, L-PRF functions primarily as a biologically active regenerative adjunct, with its modest antibacterial activity representing an additional therapeutic benefit for periodontal regenerative therapies (28–30).

Growth factors secreted from L-PRF membranes could facilitate wound healing and/or tissue repair, during which TGF-*β*1 mainly participates in the transition of wound healing from the inflammation phase to the proliferation phase and the subsequent extracellular matrix remodelling, while VEGF and PDGF-AB significantly impact angiogenesis (31, 32) and cell proliferation (33, 34). Due to the reduction in the viability of cells and the degradation of growth factors themselves after long storage periods, fresh L-PRF membranes showed advantages in terms of the sustainable release of growth factors over those who had been stored for 14 days or 28 days. VEGF was the most sensitive growth factor to storage time, lowering to only 29.5% of the fresh level after a 28-day storage. On the other hand, TGF-*β*1 could be better preserved in L-PRF membranes, detected at nearly 50% of the fresh level in cumulative release, even after a 28-day storage time. Thus, storage did not preserve growth factor release uniformly but rather resulted in differential degradation kinetics among individual growth factors, which may be explained by the different lifespan of cell components in L-PRF membranes during storage and the activation of growth factors. The stronger resilience of TGF-*β*1 against storage may be attributed to additional secretion from lymphocytes, which possess a longer life span during a static storage at 4°C (35). Furthermore, TGF-*β*1 is generally secreted in a latent form without activation, bound to latency-associated peptide (LAP) and sometimes further bound to latent TGF-*β* binding protein (LTBP), which may protect TGF-*β*1 from degradation and contributes to more stability after a longer storage period (36–38).

Among the assayed cytokines, only IL-8, IL-1b, and IL-6 were released in detectable levels, and even these were detected in only a minority of donors, indicating high inter-individual variability and resulting in limited statistical power. This heterogeneity is consistent with previous reports describing donor-dependent variation in the cytokine content of platelet concentrates, which may be influenced by platelet reactivity (39) and leukocyte composition (40–42). Donor-dependent variability in cytokine release may also be influenced by systemic conditions such as hypertension, which has been associated with a pro-inflammatory baseline state (43, 44). However, due to the limited sample size and the exploratory nature of this study, stratified comparisons between hypertensive and normotensive donors were not feasible. In addition, cellular senescence is generally associated with the development of a senescence-associated secretory phenotype (SASP), characterized by increased secretion of inflammatory mediators. However, this phenomenon was not observed in the present study, where cytokine release progressively declined during storage. One possible explanation is that the loss of metabolic activity and progressive degradation of cytokines outweighed any SASP-related secretion; however, this hypothesis requires further experimental verification. The reduction of cytokine release with storage has important implications. IL-8, IL-1b, and IL-6 are central mediators of acute inflammation, chemotaxis, and tissue repair (45, 46), and their presence in fresh L-PRF membrane may contribute to the initial recruitment of neutrophils and macrophages to the wound site, as well as the activation of resident stromal cells (42). The absence of cytokines after 14 days of storage indicates that stored L-PRF membranes may have markedly diminished pro-inflammatory signalling capacity. On the other hand, a rapid decline in inflammatory cytokine release during storage may also be advantageous in contexts where a prolonged inflammatory stimulus is undesirable (39, 40, 47).

Conditioned medium derived from both fresh and stored L-PRF membranes significantly enhanced the proliferation and migration of HOKs compared to DMEM (basic medium for preparing L-PRF conditioned medium), supporting the overall beneficial effects of the mediators released from stored L-PRF membranes. Notably, despite the decline in antibacterial activity, growth factor release, and inflammatory cytokine availability during storage, conditioned medium from L-PRF membranes stored for up to 28 days continued to stimulate HOK proliferation and migration *in vitro*. These findings suggest that keratinocyte-stimulatory activity may be relatively more resistant to storage-induced changes than other biological functions evaluated in this study. The increased metabolic activity observed in the XTT assay, which is commonly used as an indirect indicator of cell proliferation, is in agreement with previous reports attributing the biological activity of L-PRF to the gradual release of key growth factors such as PDGF, TGF-*β*1 and VEGF (48, 49). These mediators activate signalling cascades, including MAPK and PI3K/Akt, which regulate keratinocyte proliferation and survival (46). Even without statistical significance, we observed higher proliferation rates in stored L-PRF groups compared to fresh L-PRF, which may be attributed to the diminished glucose consumption by fewer viable leukocytes in stored L-PRF. From existing literature, excessive growth factors may not be necessarily advantageous for cell stimulatory processes (50) and may even be detrimental (51). Similarly, scratch assays confirmed that L-PRF membranes conditioned medium significantly promoted HOK *in vitro* wound healing. Because cell proliferation was not pharmacologically inhibited during the scratch assay, wound closure likely reflects the combined contributions of cell migration and proliferation. This finding corroborates previous evidence that platelet-derived products enhance epithelial motility via PDGF-, TGF-*β*-, and IL-6-dependent mechanisms (52, 53). The absence of significant differences between fresh and stored L-PRF suggests that stored L-PRF membranes may retain a sufficient bioactive profile to facilitate epithelial regeneration. Nevertheless, although some stored L-PRF groups exhibited slightly greater proliferative and migratory effects than fresh L-PRF, these differences were modest and generally not statistically significant. Therefore, they should not be interpreted as evidence that prolonged storage enhances the biological activity of L-PRF. Instead, these findings suggest that the regenerative potential of L-PRF may be maintained over a broader storage window than previously assumed. This maintained *in vitro* keratinocyte response should not be interpreted as evidence of preserved overall regenerative capacity either, as additional biological processes involved in tissue repair were not assessed.

Within the limitations of the present study, L-PRF membranes were obtained from relatively older healthy volunteers (mean age 60.4 years), which may limit the generalizability of the findings to younger individuals or patients with systemic conditions. In addition, L-PRF membranes from each donor were pooled during storage, meaning that analyses were performed at the donor level without assessing potential variability between individual membranes from the same donor. The agar diffusion assays in antibacterial tests cannot represent the complex characteristics of periodontal biofilms or clinical infection control. The release kinetics of growth factors and cytokines and the effects on HOK proliferation and migration were evaluated under two-dimensional *in vitro* conditions, which may not fully reflect the dynamic biological environment encountered after clinical application of L-PRF membranes. Therefore, future studies with larger cohorts should investigate the biological performance of stored L-PRF in more complex systems, such as three-dimensional oral mucosa models or *in vivo* wound healing models. Comparisons of different storage medium (e.g., saline vs. L-PRF exudate) and shorter storage intervals may also refine optimal preservation strategies.

## 5 Conclusion

Overall, these *in vitro* findings demonstrate that the biological activity of L-PRF is strongly time-dependent. Fresh L-PRF exhibited the highest cumulative release of growth factors, particularly VEGF, together with the greatest inflammatory cytokine release and antibacterial activity, especially against *P. gingivalis*. Despite these reductions during storage, L-PRF retained its ability to promote human oral keratinocyte metabolic activity and wound closure *in vitro* for up to 28 days. Further *in vivo* and clinical studies are required to determine whether these *in vitro* findings translate into improved wound healing and to define appropriate indications for the clinical use of stored L-PRF.

## Data Availability

The original contributions presented in the study are included in the article/Supplementary Material, further inquiries can be directed to the corresponding author/s.
